# Supplementary material for: Apodanthera glaziovii (Cucurbitaceae) Shows Strong Anti-Inflammatory Activity in Murine Models of Acute Inflammation
Source: Pharmaceutics. 2024 Oct 4;16(10):1298. doi: 10.3390/pharmaceutics16101298 (PMC11510368; doi:10.3390/pharmaceutics16101298)
Supplement: Supplementary file 1 [file pharmaceutics-16-01298-s001.zip › pharmaceutics-3214197-supplementary.pdf]

Supplementary material

Apodanthera glaziovii (Cucurbitaceae) Shows Strong Anti-Inflammatory Activity in Murine Models of Acute Inflammation

Maria Lorena de Oliveira Andrade, Alisson Macário de Oliveira, Pedro Artur Ferreira Marinho, Thalisson Amorim de Souza, Samuel Paulo Cibulski, Harley da Silva Alves

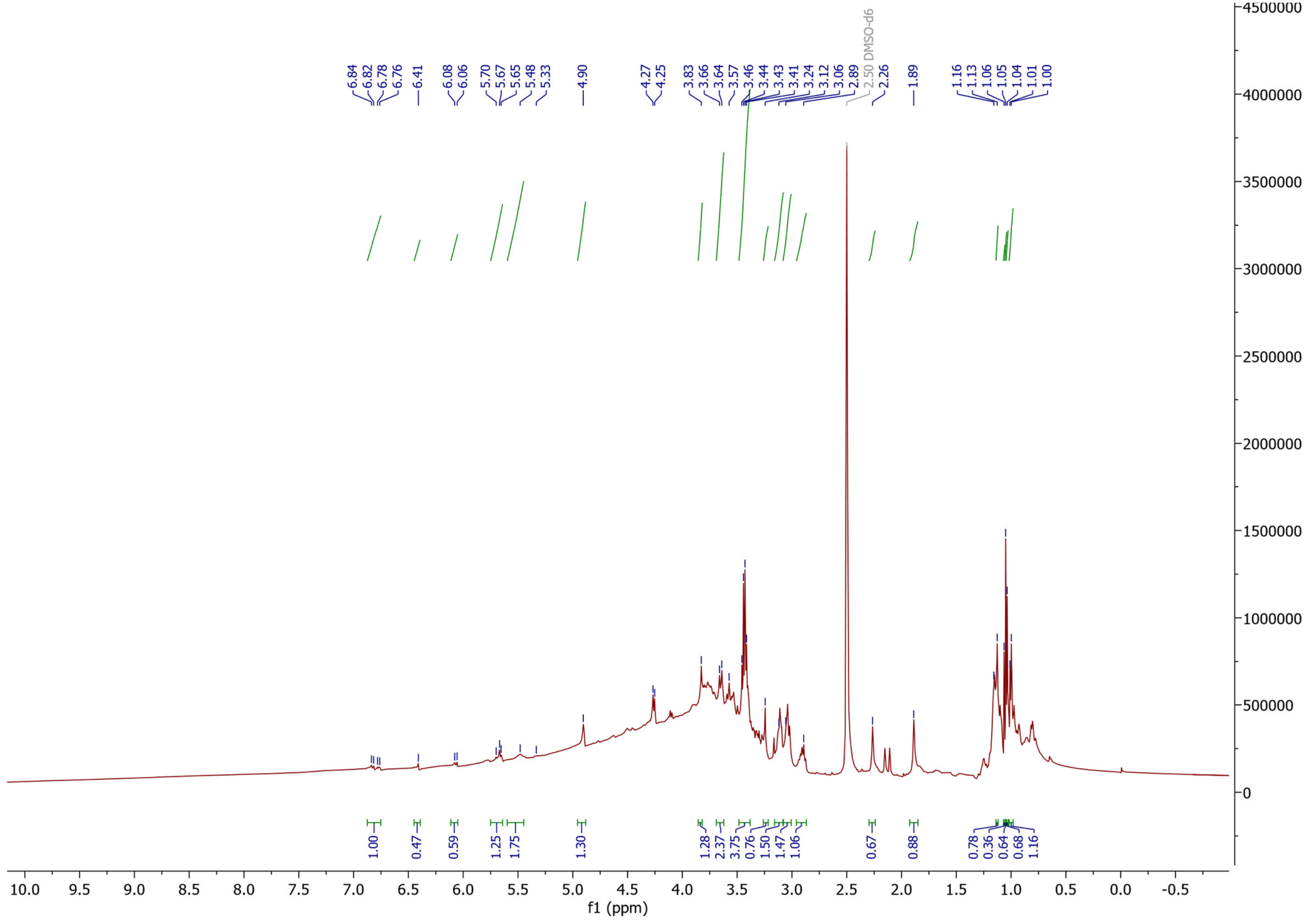

Figure S1. ^{1}**H NMR spectrum of the Stems Hydroalcoholic Extract from** ***A. glaziovii*** **(SHE-Ag) (DMSO-d_6, 500 MHz).**

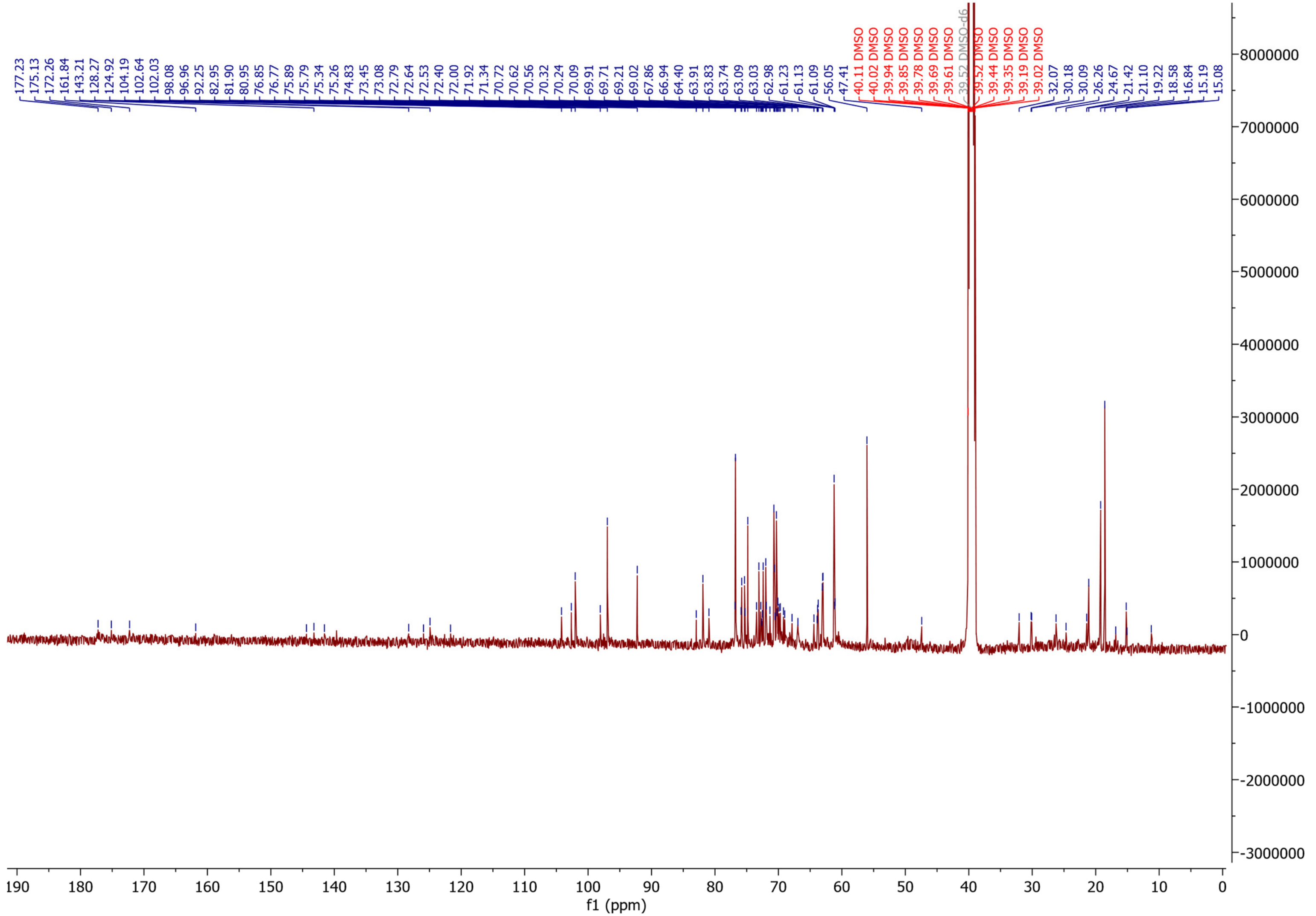

Figure S2. ^{13}**C NMR spectrum of the Stems Hydroalcoholic Extract from** ***A. glaziovii*** **(SHE-Ag) (DMSO-d$_6$, 125 MHz).**
